# Physical decoupling of XylS/*Pm* regulatory elements and conditional proteolysis enable precise control of gene expression in *Pseudomonas putida*

**DOI:** 10.1111/1751-7915.13383

**Published:** 2019-03-12

**Authors:** Daniel C. Volke, Justine Turlin, Viviënne Mol, Pablo I. Nikel

**Affiliations:** ^1^ The Novo Nordisk Foundation Center for Biosustainability Technical University of Denmark 2800 Lyngby Denmark

## Abstract

Most of the gene expression systems available for Gram‐negative bacteria are afflicted by relatively high levels of basal (i.e. leaky) expression of the target gene(s). This occurrence affects the system dynamics, ultimately reducing the output and productivity of engineered pathways and synthetic circuits. In order to circumvent this problem, we have designed a novel expression system based on the well‐known XylS/*Pm* transcriptional regulator/promoter pair from the soil bacterium *Pseudomonas putida* mt‐2, in which the key functional elements are physically decoupled. By integrating the *xylS* gene into the chromosome of the platform strain KT2440, while placing the *Pm* promoter into a set of standard plasmid vectors, the inducibility of the system (i.e. the output difference between the induced and uninduced state) improved up to 170‐fold. We further combined this modular system with an extra layer of post‐translational control by means of conditional proteolysis. In this setup, the target gene is tagged with a synthetic motif dictating protein degradation. When the system features were characterized using the monomeric superfolder GFP as a model protein, the basal levels of fluorescence were brought down to zero (i.e. below the limit of detection). In all, these novel expression systems constitute an alternative tool to altogether suppress leaky gene expression, and they can be easily adapted to other vector formats and plugged‐in into different Gram‐negative bacterial species at the user's will.

## Graphical abstract

This study describes the re‐purposing of the elements within the classical XylS/*Pm* system into a novel gene expression device which enables extremely tight expression control. The combination of the system with an extra layer of post‐translational regulation resulted in close‐to‐zero leaky expression of the target gene.

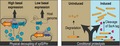

## Introduction

The soil bacterium and platform strain *Pseudomonas putida* KT2440 has become the host of choice for metabolic engineering applications that require high levels of stress resistance and a robust, versatile metabolism (Nikel *et al*., 2016; Poblete‐Castro *et al*., 2017; Nikel and de Lorenzo, 2018). A dedicated synthetic biology toolbox is key to harness the full potential of this bacterium ― and strategies for tightly controlling gene expression are not only fundamental for understanding basic properties of the cell physiology and regulatory processes thereof, but they are also crucial for biotechnological applications (Gomez *et al*., 2012; Benedetti *et al*., 2016; Chavarría *et al*., 2016; Martínez‐García and de Lorenzo, 2017; Calero and Nikel, 2019). Gene expression systems offer the possibility to control when (and how much of) a target protein should be produced. Ideally, such systems should display two states (*ON* and *OFF*), enabling to temporally separate plasmid replication and maintenance (*OFF*) from an active state, when the gene of interest (GOI) is expressed and the encoded protein(s) is/are produced (*ON*) (Ter, 2006). In addition, since the accumulation of heterologous proteins is often accompanied by metabolic burden in the recombinant cells carrying the cognate gene construct (Wu *et al*., 2016), the *ON* and *OFF* states of the expression system should be clearly differentiated and externally controllable ― that is, the system should display very low levels (ideally, zero) of basal expression (leakiness) in the non‐induced, *OFF* state.

Transferring regulatory elements of gene expression devices across bacterial hosts is often accompanied by changes in the behaviour of the system, for example basal expression, fold change in expression levels between the induced and non‐induced state, and induction dynamics (Slusarczyk *et al*., 2012; Segall‐Shapiro *et al*., 2018). Expression systems traditionally used in the model host *Escherichia coli* (e.g. LacI^Q^/P_*trc*_), for instance, are known to display different characteristics when plugged‐in into a different bacterium ― with a much higher basal expression and lower induction fold in *P. putida*. Due to the versatile metabolism of *Pseudomonas* species, rich in degradation routes for alkane and aromatic compounds (Jiménez *et al*., 2002; Nikel *et al*., 2015a,2015b), several transcriptional regulators responsive to such substrates, along with the corresponding operator sites and other regulatory elements, have been sourced from these bacterial species. Transcriptional regulator/promoter pairs of this sort include, among many others, the (alkyl)benzoate(s)‐activated XylS/*Pm* system (de Lorenzo *et al*., 1993), the alkene(s)‐activated AlkS/P_*alkB*_ system (Panke *et al*., 1999), the xylene(s)‐responsive XylR/*Pu* system (Marqués and Ramos, 1993), the phenol‐inducible DmpR regulator (Shingler and Moore, 1994) and the salicylate‐activated NahR/P_*sal*_ system (Cebolla *et al*., 1996).

The XylS/*Pm* regulatory system originates from the catabolic megaplasmid pWW0 of *P. putida* mt‐2, where it controls the transcription of genes in the lower (*meta*) cleavage pathway for degradation of aromatic compounds (Ramos *et al*., 1997). Expression systems based on the XylS/*Pm* pair have been extensively adopted for both fundamental studies and metabolic engineering of *Pseudomonas*, because of its low basal expression, relatively high fold‐change induction, and the use of low‐cost inducers such as 3‐methylbenzoate (3‐*m*Bz) and derivatives thereof (Gawin *et al*., 2017). In its native configuration, the XylS protein binds as a dimer to two operator sites (distal and proximal) closely upstream to the −35 motif of the *Pm* promoter, where it recruits the RNA polymerase with the σ^32^ or σ^38^ factors (Marqués *et al*., 1999; González‐Pérez *et al*., 2002). The dimerization is promoted by 3‐*m*Bz and related aromatic molecules, but it can also occur at high intracellular concentrations of XylS (Ruíz *et al*., 2003) ― albeit at a rate lower than that triggered by 3‐*m*Bz. Furthermore, *xylS* is under control of a constitutive promoter and a xylene‐inducible promoter (Gallegos *et al*., 1996), ensuring synchronic expression of the long upper and lower TOL degradation pathway genes through a metabolic amplification motif (Silva‐Rocha *et al*., 2011). While this transcriptional wiring is beneficial in its natural context, it hampers its application in a heterologous milieu. Zwick *et al*. (2013) showed that high *xylS* expression levels lead to increased basal *Pm* promoter activity and limited inducibility of the system by externally added effectors. Expression levels are likewise dependent on gene dosage: while the system is usually tightly controlled as a single‐copy genomic integration, basal expression dramatically increases with the copy number if the regulatory elements are plasmid‐borne. This situation helps explaining the significant discrepancies in the induction levels reported for the XylS/*Pm* system, ranging from ~10‐fold for medium copy number plasmids based on the origin of vegetative replication (*oriV*) pBBR1 in *P*. *putida* (Calero *et al*., 2016) to ~100‐fold for low copy number plasmids with *oriV*(RK2) in *E. coli* (Winther‐Larsen *et al*., 2000). The origins of replication commonly used in *Pseudomonas* display medium‐to‐high copy number levels [*oriV*(pBBR1) = 30 ± 7; *oriV*(RK2) = 20 ± 10; *oriV*(RSF1010) = 130 ± 40; and *oriV*(pRO1600/ColE1) ~30–40 (Jahn *et al*., 2016; Cook *et al*., 2018)].

**Figure 1:**
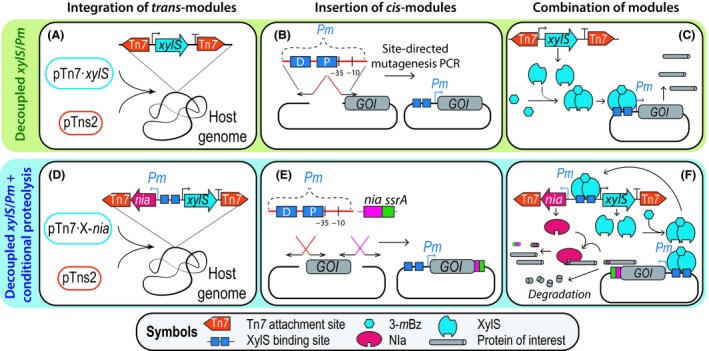
Scheme of tightly regulated expression systems for *Pseudomonas* based on physically decoupled XylS/*Pm* elements and conditional proteolysis.A. The *trans*‐module of the system, carrying the *xylS* regulator gene under transcriptional control of its native promoters, is flanked by the Tn*7* recognitions sites borne by plasmid pTn*7*·*xylS*. The *trans*‐module is integrated into the host genome through the Tn*7* transposase functions contained in the helper plasmid pTns2.B. Insertion of the *cis*‐module of the system into the vector of interest. The *cis*‐module is comprised of the distal and proximal XylS‐binding sites, followed by the −35 and −10 binding sites for the RNA polymerase. The whole module is 70‐bp long and can be easily introduced into any target vector through site‐directed mutagenesis PCR.C. A suitable plasmid carrying the *cis*‐module is delivered into a host *Pseudomonas* strain already containing the *trans*‐module integrated into the chromosome. The genomic integration of *xylS* in monocopy ensures low transcription levels of the regulator gene; upon addition of 3‐*m*Bz as the inducer, XylS dimerizes and activates transcription from the *Pm* promoter.D. The *trans*‐module carrying the gene encoding the NIa protease under the transcriptional control of XylS/*Pm* is flanked by the Tn*7* recognition sites borne by plasmid pTn*7*·X‐*nia*. This module is integrated into the host genome through the Tn*7* transposase functions contained in the helper plasmid pTns2.E. Insertion of the *Pm* promoter and the 3′‐tag for conditional proteolysis to a gene of interest.F. The constructed plasmid (carrying the *cis*‐module) is delivered into a host *Pseudomonas* strain already containing the *trans*‐module in the chromosome. The inducer 3‐*m*Bz promotes dimerization of XylS and, consequently, triggers transcription from the *Pm* promoter. Note that the *Pm* promoter drives the transcription of the gene of interest as well as that of *nia*. Only if both gene products are present in sufficient amounts, they will interact and the target protein is then relieved from the proteolysis tag – thereby enabling stable accumulation of the product. The abbreviations used in this scheme are as follows: 3‐*m*Bz, 3‐methylbenzoate; *GOI*, gene of interest; NIa, nuclear inclusion protein A.

In order to enable better control of gene expression in Gram‐negative bacteria (and, in particular, in *Pseudomonas* species), in this study, we have re‐wired the functional elements of the XylS/*Pm* regulatory system (Fig. 1) in combination with the recently described *FENIX* system for post‐translational control (Durante‐Rodríguez *et al*., 2018). By physically decoupling the *xylS* and *Pm* components, while rendering target protein(s) sensitive to conditional proteolysis, we have achieved a tightly controlled expression output of the system, with close‐to‐zero leakiness and in a plasmid copy number ― independent fashion.

## Protocol

### Materials

- *Sucrose solution:* 300 mM sucrose (cat. # 84100; Sigma‐Aldrich Corp., St. Louis, MO, USA) in double‐distiled water (_dd_H_2_O). Sterilized by filtration and kept at room temperature.
- *Induction solution:* 0.5 M 3‐*m*Bz (cat. # M29908; Sigma‐Aldrich Corp.) in _dd_H_2_O, pH adjusted to neutrality by dropwise addition of 5 M NaOH until no 3‐*m*Bz precipitation is visible. Stored at room temperature.
- *Lysogeny broth (LB) medium:* 10 g l^−1^ tryptone, 5 g l^−1^ yeast extract, and 5 g l^−1^ NaCl; dissolved in _dd_H_2_O and autoclaved. Bacteriological agar (cat. # A5306; Sigma‐Aldrich Corp.) was added at 15 g l^−1^ for LB agar plates before autoclaving.
- *Kanamycin (Km) stock solution:* 50 mg ml^−1^ Km sulfate (cat. # T832.3; Carl Roth GmbH & Co. KG, Karlsruhe, Germany) dissolved in _dd_H_2_O, sterile filtered and stored at −20°C. Used at a final concentration of 50 μg ml^−1^.
- *Gentamycin (Gm) stock solution:* 10 mg ml^−1^ Gm sulfate (cat. # G1264; Sigma‐Aldrich Corp.) in _dd_H_2_O, sterile filtered and stored at −20°C. Used at a final concentration of 10 μg ml^−1^.

### General procedures

- *Plasmid purification:* The *Nucleospin Plasmid EasyPure* Kit (cat. # 740727.250; Macherey‐Nagel GmbH & Co. KG, Düren, Germany) was used for plasmid purification according to the manufacturer's instructions.
- *Site‐directed mutagenesis:* 45 μl of the PCR product is treated with DpnI (cat. # FD1703; Thermo Fisher Scientific, Waltham, MA, USA) according to the manufacturer's instructions to digest the template DNA used for the amplifications. An aliquot of 7 μl of digested PCR product is incubated with 1 μl of T4 polynucleotide kinase (cat. # EL0014; Thermo Fisher Scientific), 1 μl of T4 DNA ligase (cat. # EK0031; Thermo Fisher Scientific) and 1 μl of ligation buffer for 2 h at room temperature. The total amount of 10 μl is then used for transforming chemically competent *E. coli* DH5α cells (Boyer and Roulland‐Dussoix, 1969; Ruiz *et al*., 2006).
- *PCR reaction conditions: Phusion* Hot Start II DNA polymerase (cat. # F549L; Thermo Fisher Scientific) was used according to the manufacturer's instructions with HF‐buffer in the presence of 3% (v/v) DMSO (cat. # F‐515; Thermo Fisher Scientific).
- *Agarose gel electrophoresis:* 5 μl of the solution containing the DNA fragment(s) to be analysed was mixed with 1 μl of gel loading dye (cat. # R0611; Thermo Fisher Scientific) and analysed on a 1% (w/v) agarose gel (cat. # BN‐50004; BioNordika Denmark A/S, Herlev, Denmark) in TAE buffer [1 mM EDTA (cat. # E6758; Sigma‐Aldrich Corp.), 40 mM Tris (cat. # T1503; Sigma‐Aldrich Corp.) and 20 mM acetic acid (cat. # 33209; Sigma‐Aldrich Corp.)]. Fragment sizes were compared against a DNA ladder standard run in parallel (cat. # SM0313; Thermo Fisher Scientific).

### Technical implementation

In this study, we have implemented two approaches to tightly control gene expression in Gram‐negative bacteria, and in particular *P*. *putida*, by rewiring the transcriptional machinery of the well‐characterized XylS/*Pm* expression system. Both procedures rely on the physical separation of the gene encoding the transcriptional regulator and the *Pm* promoter, as indicated below.

#### Genomic integration of a *trans*‐module encoding the XylS transcriptional regulator and the NIa protease in *P*. *putida* KT2440

The starting point for the construction of the novel gene expression device was to physically separate the gene encoding the XylS transcriptional regulator and its cognate promoter (Fig. 1). On this background, a *trans*‐module was defined herein as any genomic part that is physically separated from the GOI. Likewise, in the devices presented in this study, the *trans*‐modules are DNA segments integrated into the genome of *P*. *putida* KT2440 (i.e. *xylS* or *xylS*/*Pm*→*nia*). Note that this genetic architecture contrasts the traditional arrangement of most expression systems, that is, a plasmid‐based XylS/*Pm* system in which the parts are encoded in the same DNA molecule. Accordingly, *cis*‐modules are adjacent to the GOI (i.e. the *Pm* promoter and the synthetic *nia/ssrA* tag). The genomic integration of the *xylS* or *xylS*/*Pm*→*nia* modules was accomplished by co‐electroporating plasmids pTn*7*·*xylS* or pTn*7*·X‐*nia* (Table 1) respectively, together with the helper plasmid pTns2 (which encodes the Tn*7* transposase functions) into wild‐type *P. putida* KT2440. For this, a 10‐ml overnight culture of *P. putida* KT2440, grown in LB medium in a 50‐ml tube (Corning™, cat. # 352070; Thermo Fisher Scientific), was centrifuged at 4000 *g* for 5 min at room temperature. The cell pellet was washed by resuspension in 1 ml of 300 mM sucrose and transferred to a 2‐ml reaction tube followed by centrifugation at 15 000 *g* for 1 min. The supernatant was carefully discarded, and the washing steps were repeated twice. In the final step, the cell pellet was resuspended in 0.4 ml of 300 mM sucrose. An aliquot of 0.1‐ml of the resulting suspension was mixed with 200 ng of each plasmid and transferred to a 0.2‐cm gap electroporation cuvette (cat. # 165‐2086; Bio‐Rad Corp., Hercules, CA, USA; Smith and Iglewski, 1989). A single 2.5‐kV electric pulse was applied for up to 6.0 ms in a microPulser electroporator (Bio‐Rad Corp.), followed by adding immediately 1 ml of LB medium. Cells were recovered by incubating the culture at 30°C with rotational agitation for 2 h. The biomass was then concentrated in 0.1 ml by centrifugation as indicated above and plated onto LB agar plates supplemented with Gm. Gm‐resistant (Gm^R^) colonies were checked by colony PCR for the correct insertion of the Tn*7* module with oligonucleotides Gm_check‐F and Gm_check‐R (Table 2), which anneal within the Tn*7* integration site‐adjacent gene *PP_5408* and in the left transposon‐flanking site (Tn*7*L) respectively. The correct insertion of the Gm^R^‐determinant yields a 400‐bp amplification fragment (Zobel *et al*., 2015). Selected clones, which display the correct fragment, were further subjected to sequencing with the primer pair pS1 and pS2 (Table 2). Alternatively, plasmids can be delivered into *P. putida* by triparental mating; a detailed procedure is given by Zobel *et al*. (2015). These operations yielded strains KT·P and KT·PN, in which the *xylS* or *xylS*/*Pm*→*nia* modules respectively, are stably inserted in the att·Tn*7* site in the chromosome of *P*. *putida* KT2440 (Fig. 1).

**Table 1:** Plasmids used in this work

Plasmid name^a^	Relevant characteristics^b^	Source or reference
pTn*7*‐M	Tn*7* integration vector; *oriV*(R6K); Km^R^, Gm^R^	Choi *et al*. (2005)
pTn7·*xylS*	Derivative of vector pTn*7*‐M used for chromosomal integration of *xylS*	This work
pTn7·X‐*nia*	Derivative of vector pTn*7*‐M used for chromosomal integration of *xylS/Pm*→*nia*	This work
pTns2	Helper plasmid constitutively expressing the *tnsABCD* genes encoding the Tn*7* transposase; *oriV*(R6K); Amp^R^	Choi *et al*. (2005)
pSEVA238	Expression vector; *oriV*(pBBR1), XylS/*Pm* expression system; Km^R^	Silva‐Rocha *et al*. (2013)
pSEVA248	Expression vector; *oriV*(pRO1600/ColE1), XylS/*Pm* expression system; Km^R^	Silva‐Rocha *et al*. (2013)
pS238·NIa	Derivative of vector pSEVA238 used for regulated expression of *nia*, encoding the potyvirus NIa protease; XylS/*Pm*→*nia*; Km^R^	Durante‐Rodríguez *et al*. (2018); B. Calles and V. de Lorenzo (unpublished data)
pS238·GFP	Derivative of vector pSEVA238 used for regulated expression of *msfGFP*; XylS/*Pm*→*msfGFP*; Km^R^	This work
pS248·GFP	Derivative of vector pSEVA248 used for regulated expression of *msfGFP*; XylS/*Pm*→*msfGFP*; Km^R^	This work
pSEVA237M	Cloning vector; *oriV*(pBBR1); promoter‐less *msfGFP*; Km^R^	Silva‐Rocha *et al*. (2013)
pSEVA247M	Cloning vector; *oriV*(pRO1600/ColE1); promoter‐less *msfGFP*; Km^R^	Silva‐Rocha *et al*. (2013)
pS23·*Pm*‐GFP	Derivative of vector pSEVA237M bearing *Pm*→*msfGFP*; Km^R^	This work
pS24·*Pm*‐GFP	Derivative of vector pSEVA247M bearing *Pm*→*msfGFP*; Km^R^	This work
pS23·*Pm*‐GFP*^c^	Derivative of vector pSEVA237M bearing *Pm*→msfGFP*; Km^R^	This work
pS24·*Pm*‐GFP*	Derivative of vector pSEVA247M bearing *Pm*→msfGFP*; Km^R^	This work

a. Plasmids can be obtained from Addgene (http://www.addgene.org) with the following deposit numbers: pTn7·*xylS* (122591), pTn7·X‐*nia* (122592), pS23·*Pm*‐GFP (122593) and pS24·*Pm*‐GFP (122594).

b. *Antibiotic markers*: Amp, ampicillin; Gm, gentamicin; Km, kanamycin.

c. Conditionally proteolizable variants of msfGFP are indicated by an asterisk (*) symbol.

**Table 2:** Oligonucleotides used in this work.^a^

Oligonucleotide	Sequence (5′→3′)	*T*_m_ (°C)	Use
*Pm*_ins‐F	TAT CTC TAG TAA GGC CTA CCC CTT AGG CTT TAT GCA AGC TTA GGA GGA AAA ACA TAT GCG	60	Insertion of *Pm* into vector pSEVA237M by site‐directed mutagenesis PCR
*Pm*_ins‐R	GCC ATT TTT TGC ACT CCT GTA TCC GCT TCT TGC AAT TAA TTA AAG GCA TCA AAT AAA ACG AAA GGC TCA	59	
*ssr_nia*‐F	CGC TAA CGA CGA TAA CTA CGC CCT GGC TGC GTA AAC TAG TCT TGG ACT CCT GTT GAT AGA	60	Insertion of a *nia/ssrA* tag into pS23·Pm‐GFP by site‐directed mutagenesis PCR
*ssr_nia*‐R	GCA CGT TCA TCA GCT TGA TGC ACC ACG ACG TTG GAC TCG CCT TTG TAG AGT TCA TCC ATG CCG TGC	57	
*xylS*·pTn*7*‐F†	ACG TCT TAA UTA AAC GTT CGT AAT CAA GCC ACT T	61	Amplification of *xylS* to clone into vector pTn7‐M
*xylS*·pTn*7*‐R†	AGG ACA CUG CAC TTT ATG CTG GTT ATG C	60	
pTn*7*·*xylS*‐F†	AGT GTC CUA GGC CGC GGC CGC	63	Amplification of pTn7‐M to insert *xylS or xylS*/*Pm*→*nia*
pTn*7*·*xylS*‐R†	ATT AAG ACG UCT TGA CAT AAG CCT GTT CGG TTC	62	
pTn*7*·*nia*‐F†	ACT CAG GGU ACC CGG GGA TCC TCT AGA	58	
*nia*·pTn*7*‐R†	ACC CTG AGU GTA AAC AAA TTC CCC ATC AAG A	57	Cloning of *xylS*/*Pm*→*nia*
Gm_check‐F	AGT CAG AGT TAC GGA ATT GTA GG	55	Checking insertion of Gm^R^ into the chromosome
Gm_check‐R	ATT AGC TTA CGA CGC TAC ACC C	56	
*gfp*_RBS‐F	AAT CCT AGG CCG CGA CGC ATG TTT AGG AGG AAA AAC ATA TGC GTA AAG GTG AAG AAC TGT	63	Cloning of *msfGFP*
*gfp*‐R	AAG ACT AGT CAT TTA TTT GTA GAG TTC ATC CAT G	54	
pS1	AGG GCG GCG GAT TTG TCC	60	Check SEVA plasmids cargo
pS2	GCG GCA ACC GAG CGT TC	59	

a. Oligonucleotides designed for *USER* assembly are indicated with a † symbol.

#### Design and construction of a set of standard vectors containing the *Pm* promoter

**Figure 2:**
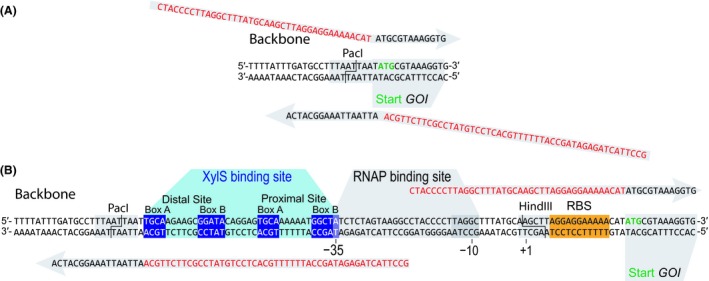
Standard procedure used for easy insertion of the *Pm* promoter into any plasmid backbone.A. Insertion of the *Pm* element into a vector by site‐directed mutagenesis PCR. Each primer anneals to the plasmid backbone through its 3′‐end (shown in black), while the 5′‐end carries part of the *Pm* region (shown in red).B. Structure of the *Pm* promoter element. The promoter contains two XylS binding sites, depicted as distal and proximal binding site. The σ factors σ^32^ and σ^38^ bind and recruit the RNA polymerase into the –35 and –10 motifs of the promoter (González‐Pérez et al., 2002; Domínguez‐Cuevas et al., 2005). Direct cloning this region upstream of the gene of interest is achieved either by site‐directed mutagenesis PCR (necessary primers are shown in grey), restriction/ligation (relevant restriction sites, compatible with the *Standard European Vector Architecture*, are indicated), or a different cloning technique (e.g. *USER* or Gibson assembly).

A synthetic *cis*‐module, carrying the essential *Pm* region (which includes the XylS‐ and σ factor‐binding sites), was constructed in such a way that it was made compatible with different cloning strategies ― so that the GOI can be inserted into any plasmid containing the module. This objective can be achieved, for example by traditional restriction/ligation cloning using the HindIII and SpeI sites present in plasmid pS23·*Pm*‐GFP (Table 1) to replace the gene encoding the monomeric superfolder GFP (msfGFP) with any other GOI. Otherwise, the synthetic *cis*‐module spanning the promoter region can be inserted into a different plasmid already harbouring the GOI. Such insertion can be achieved by site‐directed mutagenesis PCR with a set of primers containing the *Pm* motif (Fig. 2A). Forward and reverse oligonucleotides (*Pm*_ins‐F and *Pm*_ins‐R, Table 2) were designed so that they amplify the flanking DNA regions surrounding the locus intended for insertion of *Pm via* an extension added to their 5′‐termini. Thus, the 5′‐end of the forward primer anneals immediately in front of the intended insertion site, whereas the sequence complementary to the GOI is placed at its 3′‐end. For this purpose, the 5′‐terminus of the forward primer is appended with the synthetic sequence 5′‐CTA CCC CTT AGG CTT TAT GCA AGC TTA GGA GGA AAA ACA T‐3′. Following the same reasoning, the reverse primer is extended at its 5′‐end with the synthetic sequence 5′‐GCC TTA CTA GAG ATA GCC ATT TTT TGC ACT CCT GTA TCC GCT TCT TGC A‐3′ (Fig. 2B). Following the amplification of the target plasmid with these primers, a 5‐μl aliquot of the PCR product was analysed by gel electrophoresis. If the fragment had the correct size (i.e. length of the plasmid), the remaining PCR product was used according to the site‐directed mutagenesis PCR protocol as indicated in the previous section and then transformed into chemically competent *E. coli* DH5α cells (cat. # 18265017; Thermo Fisher Scientific). The efficiency of this procedure was very high, and the plasmid of three different colonies could be immediately sequenced without previous confirmation by colony PCR.

#### Tagging proteins of interest with a synthetic NIa/SsrA degradation tag for conditional proteolysis

**Figure 3:**
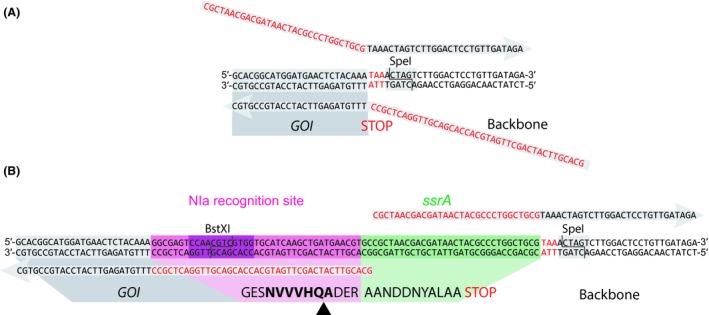
Introduction of the hybrid NIa/SsrA degradation tag into a target protein.A. The degradation tag can be fused to a gene of interest by site‐directed mutagenesis PCR. Primers used to fuse the tag to the monomeric superfolder GFP (msfGFP) in plasmid pS23·*Pm*‐GFP* are shown in dark grey. The 3′‐terminus of the primers anneals to the plasmid (black letters) and primes amplification of the fragment. The 5′‐ends carry the synthetic NIa/SsrA tag proper (red letters).B. After re‐circularization of the PCR product, the resulting plasmid contains the NIa/SsrA tag fused to the gene of interest. The tag includes the conserved NIa recognition site (indicated in boldface) with its cleavage site (black triangle) followed on the 3′‐terminal by the predicted SsrA degradation signal from *P. aeruginosa* (Flynn et al., 2001). Alternatively, other cloning strategies can be used to insert the degradation tag for conditional proteolysis, such as restriction/ligation (relevant restriction sites, compatible with the *Standard European Vector Architecture*, are indicated), or a different cloning technique (e.g. *USER* or Gibson assembly).

The hybrid *nia/ssrA* tag can be inserted at the 3′‐end of the GOI either through restriction/ligation cloning or site‐directed mutagenesis PCR. Alternatively, the proteolytic tag can be directly inserted into another plasmid by using the restriction sites BstXI and SpeI. To introduce the synthetic tag sequence *via* PCR, oligonucleotides were designed to include the *nia/ssrA* moiety. The reverse primer was designed against the 3′‐end of the GOI (but excluding the *STOP* codon), while the forward primer included both the *STOP* codon and a sequence complementary to the plasmid backbone (Fig. 3A). The 5′‐terminus of the forward primer was appended with the sequence 5′‐CGC TAA CGA CGA TAA CTA CGC CCT GGC TGC G‐3′, while the reverse primer was extended at its 5′ end with the sequence 5′‐GCA CGT TCA TCA GCT TGA TGC ACC ACG ACG TTG GAC TCG CC‐3′ (Fig. 3B). These sequences were added to insert the synthetic *nia/ssrA* tag, which contains the conserved NIa recognition site (NVVVHQ•A, the cleavage site is indicated with the • symbol; García *et al*., 1989) and the SsrA degradation tag, designed according to the conserved SsrA recognition sequence described for *P. aeruginosa* (Flynn *et al*., 2001). Following the amplification of the target plasmid with these primers, an aliquot of the PCR product (5 μl) was analysed by gel electrophoresis and, if the correct fragment size was observed, the remainder of the reaction was used for site‐directed mutagenesis PCR as indicated in the preceding section. Chemically competent *E. coli* DH5α cells were transformed with the reaction mix as indicated previously. The efficiency of this procedure resulted to be very high, and plasmid DNA from three different colonies can be immediately sequenced without previous confirmation by colony PCR.

#### Parallel integration of the *Pm* promoter and a synthetic NIa/SsrA degradation tag for conditional proteolysis

In order to obtain a construct containing the *Pm* promoter and the GOI fused to the tag in one step, the gene encoding msfGFP can be replaced by the GOI in plasmid pS23·*Pm*‐GFP* or pS24·*Pm*‐GFP* (which differ in the *oriV* and hence in plasmid copy number; Table 1) through traditional restriction/ligation using the restriction sites HindIII and BstXI. In order to introduce both sites in another plasmid, *USER* assembly (Cavaleiro *et al*., 2015) or Gibson assembly (Gibson *et al*., 2009) can be likewise used as desired.

## Application example

After observing high basal expression stemming from the XylS/*Pm* system in plasmid‐based systems, we set out to characterize this expression system on different standard vectors (i.e. carrying different *oriV* and hence displaying diverse copy numbers) to gain insight in the regulation of the transcriptional response. In order to investigate the consequences of copy number on the behaviour of the XylS/*Pm* system, plasmids with different copy numbers were constructed (Table 1), containing the gene encoding msfGFP under the transcriptional control of XylS/*Pm*. Plasmids pS238·GFP and pS248·GFP were constructed following the *Standard European Vector Architecture* (SEVA; Silva‐Rocha *et al*., 2013; Martínez‐García *et al*., 2014) by amplification of *msfGFP* from pSEVA237M (Table 1) with the primer pair *gfp*_RBS‐F and *gfp*‐R (Table 2) and ligation of the DNA fragment into the SpeI and AvrII‐digested vectors pSEVA238 and pSEVA248. The msfGFP fluorescence (λ excitation = 485 nm, λ emission = 516 nm) was determined during cultivation of the cells in M9 minimal medium (Nikel *et al*., 2015a,2015b) supplemented with 0.2% (w/v) citrate at 30°C in a 96‐well plate (Synergy H1, BioTek). The expression strength of *msfGFP* was estimated from the change of fluorescence divided by the change of optical density at 600 nm.

Interestingly, higher copy number correlated with elevated maximum expression of *msfGFP* under the XylS/*Pm* system, but inducibility (i.e. the fold‐change difference between the induced and uninduced state) also decreased drastically. In particular, we observed a nine and threefold change in the ratio of induced/uninduced states when *P*. *putida* was expressing *msfGFP* from plasmid pS238·GFP or pS248·GFP respectively. This result is in accordance to the functional model of the XylS regulator proposed by Zwick *et al*. (2013), indicating that the formation of XylS dimers depends on inducer concentration at low XylS concentration, while at higher XylS availability, active regulator dimers can be formed even without inducer. Furthermore, this model points that the active XylS dimer concentration seems to be limited by its low solubility in the cell cytoplasm.

Against this background, and in order to uncouple the copy number of *xylS* (hence, the intracellular concentration of XylS) from *msfGFP* under the transcriptional control of *Pm*,* xylS* was integrated into the chromosome by using the Tn*7*‐bearing plasmid pTn*7*·*xylS*. For the construction of plasmid pTn*7*·*xylS*, the fragment contain *xylS* was amplified from plasmid pS238·*nia* with the primer pair *xylS*·pTn*7*‐F and *xylS*·pTn*7*‐R (Table 2), and vector pTn*7*‐M was amplified with the primer pair pTn*7*·*xylS*‐F and pTn*7*·*xylS*‐R. These DNA fragments were then merged in a *USER* assembly reaction. Furthermore, plasmids pS23·*Pm*‐GFP and pS24·*Pm*‐GFP (which contain *msfGFP* under the control of *Pm*, but do not carry *xylS*) were constructed in parallel. This set of plasmids were obtained by site‐directed mutagenesis PCR of plasmids pSEVA237M and pSEVA247M with the primers *Pm*_ins‐F and *Pm*_ins‐R (Table 2). A *P. putida* strain, carrying *xylS* integrated into the genome, and transformed with either plasmid pS23·*Pm*‐GFP (in which the *oriV* is pBBR1) or pS24·*Pm*‐GFP (in which the *oriV* is RO1600/ColE1) showed inducibility levels of 170 and 84‐fold when compared to control experiments without 3‐*m*Bz respectively. In these uncoupled systems, the basal expression strength decreased more than the induced expression strength. On the other hand, it was observed that the maximum expression strength decreased, likely because XylS is produced at subsaturation concentrations with respect to activation of *Pm*. It is also possible that the basal expression of the GOI decreased due to a lower amount of available XylS (which would dimerize without inducer if its concentration reaches a certain threshold). Even though the uncoupling strategy led to a drastic improvement in the inducibility of the XylS/*Pm* expression system, some degree of basal (i.e. leaky) expression was still detected.

In order to reduce basal expression further, we set to design a system in which not only *xylS* and *Pm* copy numbers are controlled by physical uncoupling, but also expression of the GOI was further controlled by post‐translational degradation control. Plasmids pS23·*Pm*‐GFP* and pS24·*Pm*‐GFP* were constructed through site‐directed mutagenesis PCR using plasmids pS23·*Pm*‐GFP and pS24·*Pm*‐GFP respectively, as the template and primers *ssr*_*nia*_F and *ssr*_*nia*‐R (Table 2). The msfGFP used in our experiments appears to be an excellent reporter for post‐translational control, as it is very stable and therefore tends to accumulate in the cells (Pedelacq *et al*., 2006). The gene encoding the nuclease NIa was integrated through the use of the plasmid pTn*7*·X‐*nia*. For the construction of plasmid pTn*7*·X‐*nia*, the fragment containing *xylS/Pm*→*nia* was amplified from plasmid pS238·NIa with the primer pair *xylS*·pTn*7*‐F and *nia*·pTn*7*‐R (Table 2), and plasmid pTn*7*‐M was amplified with the primer pair pTn*7*·*nia*‐F and pTn*7*·*xylS*‐R. These fragments were then merged in a *USER* assembly reaction as indicated above.

**Figure 4:**
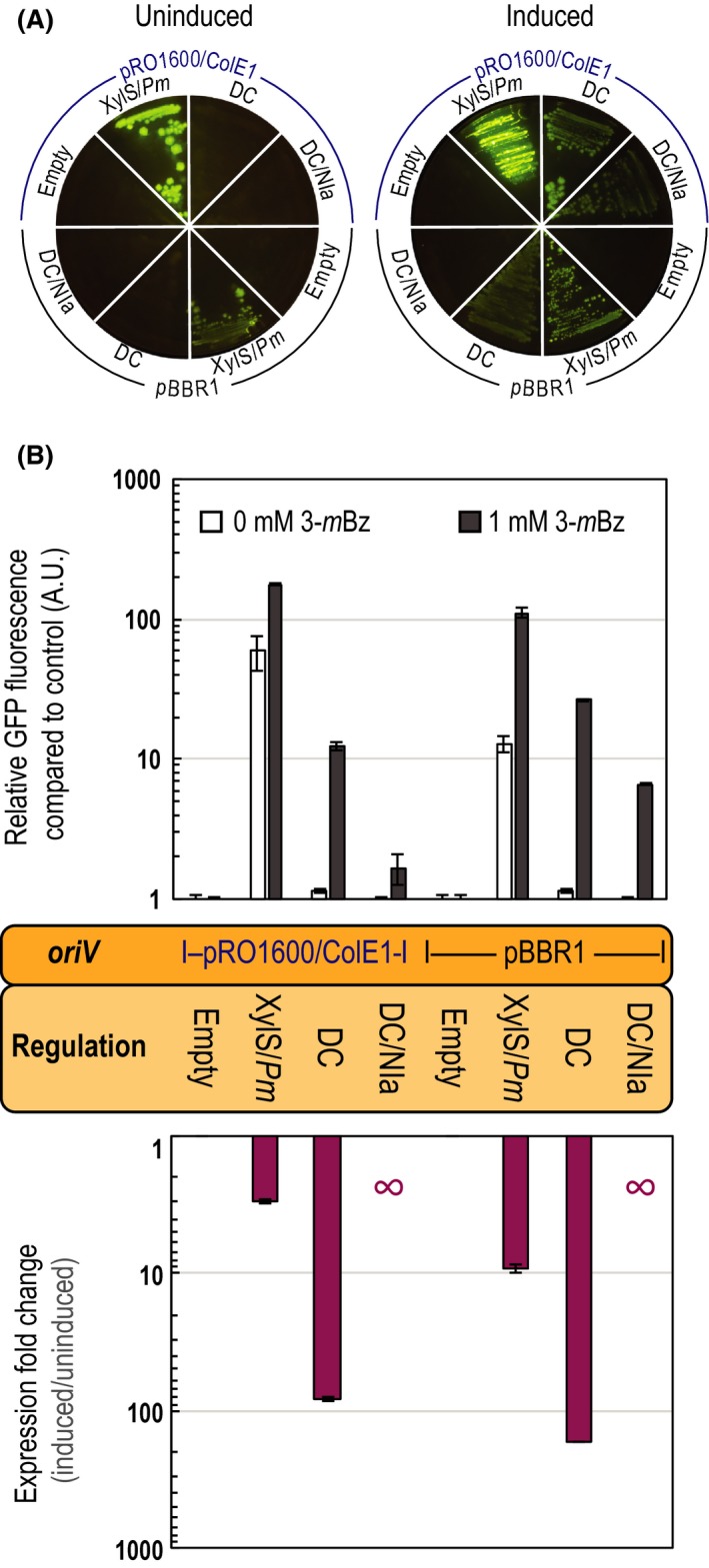
Characterization of the tightly regulated expression systems for *Pseudomonas* based on physically decoupled XylS/*Pm* elements and conditional proteolysis.A. M9 minimal medium plates with 0.2% (w/v) citrate, containing 0 mM (left) or 1 mM (right) 3‐methylbenzoate (3‐*m*Bz) were seeded with *P*. *putida* with an empty Tn*7* module integrated into the chromosome and transformed with either vector pSEVA231 or vector pSEVA241, which served as negative controls (indicated as Empty in the figure). The same strain was transformed either with plasmid pS238·GFP or pS248·GFP carrying the XylS/*Pm* expression system in its original configuration (indicated as XylS/*Pm* in the figure). Strain KT·P, containing a chromosomal copy of *xylS*, was transformed either with plasmid pS23·*Pm*‐GFP or pS24·*Pm*‐GFP, in which the *xylS*/*Pm* system has been physically decoupled (indicated as DC in the figure). Strain KT·PN, containing a single *xylS/Pm*→*nia* integration in the chromosome, was transformed either with plasmid pS23·*Pm*‐GFP* or pS24·*Pm*‐GFP* harbouring the decoupled *xylS*/*Pm* system and equipped with conditional proteolytic control of GFP due to inducible *nia* expression (indicated as DC/NIa in the figure). Strains harbouring plasmids pS24x (x represents any of the variants described above) carrying the origin of vegetative replication (*oriV*) pRO1600/ColE1 were seeded in the upper part of the plate, while strains harbouring the plasmids pS23x, carrying *oriV*(pBBR1), were seeded in the lower part. Plates were incubated at 30°C for 18 h. The plates were visualized under blue light (Safe‐Imager, Invitrogen) and photographed.B. The increase in msfGFP fluorescence per unit of biomass (estimated as the optical density at 600 nm) relative to the negative control was measured during cultivation in M9 minimal medium containing 0.2% (w/v) citrate supplemented or not with 3‐*m*Bz at 1 mM (upper part) and the resulting expression fold changes (lower part). Depicted in the diagram are the genetic elements either integrated into the single Tn*7* integration site in the chromosome or plasmid‐borne. Bars represent the mean values of the corresponding parameter ± standard deviation (*n *≥* *3 in all cases), and a Student *t*‐test revealed significant differences between all groups. Note that in some cases the level of msfGFP fluorescence of control experiments was below the limit of detection; in these experiments (DC/NIa), the ratio between induced and uninduced conditions is indicated with a ∞ symbol.

The expression system employing transcriptional and post‐translational control over the expression of msfGFP showed lower expression levels when induced as compared with the expression system without post‐translational control (Fig. 4). This phenomenon could be caused by the presence of additional XylS binding sites (in the *Pm* promoter of the integration), which recruit active XylS dimers and hence lowers the actual concentration of free XylS dimers, which can then bind to the plasmid‐borne XylS binding sites. Interestingly, no basal expression was observed with either plasmid, which leads to close‐to‐zero levels of leaky expression in the absence of 3‐*m*Bz and, at the same time, yielded the highest (indicated as ∞ in Fig. 4B) inducibility of the system.

## Discussion

In this work, we have demonstrated how the repurposing of a classical gene expression system by means of the physical separation of *xylS* and *Pm* can lead to much higher induction fold changes compared with the systems carrying *xylS* and *Pm* on the same vector. The phenomenon underlying the results observed in our uncoupled system could be connected to the data reported by Goñi‐Moreno *et al*. (2017), indicating that the proximity of a source (i.e. XylS) and target (i.e. *Pm* promoter) elements dictates noise patterns of the expression system. Furthermore, the dual transcriptional and post‐translational control of the gene expression flow in our system reduced basal expression down to undetectable amounts of msfGFP caused by leaky expression of the GOI thereof. Since the expression of *xylS* might be too low to reach saturation of the *Pm* promoter by active XylS dimers upon addition of the chemical inducer, the final output of the system was lower than in the plasmid‐based counterpart. If stronger gene expression is desired, the system could be further improved through the implementation of engineered *xylS* and/or variants of the *Pm* promoter (Bakke *et al*., 2009; Vee Aune *et al*., 2010; Zwick *et al*., 2012). In any case, for applications where tight control over gene expression is crucial, the modular expression systems presented here offer a solid alternative that can be easily implemented into virtually any combination of vectors and GOIs (and Gram‐negative bacterial hosts). In particular, the synthetic systems discussed in this article constitute a valuable tool for maintaining low leaky expression of genes involved in pathways leading to toxic intermediates or products (a classical problem in metabolic engineering), and for the design of circuits in which the transcriptional output of a given system is ‘cascaded’ into another signal, thereby multiplying the tightness of the transcriptional regulation thereof.

## Conflict of interest

None declared.
